# Barley grain for ruminants: A global treasure or tragedy

**DOI:** 10.1186/2049-1891-3-22

**Published:** 2012-07-09

**Authors:** Akbar Nikkhah

**Affiliations:** 1 Department of Animal Sciences, Faculty of Agricultural Sciences, University of Zanjan, Zanjan, 313-45195, Iran

**Keywords:** Barley, Cereal, Ruminant, Starch, Treasure

## Abstract

Barley grain (*Hordeum vulgare L.*) is characterized by a thick fibrous coat, a high level of ß-glucans and simply-arranged starch granules. World production of barley is about 30 % of that of corn. In comparison with corn, barley has more protein, methionine, lysine, cysteine and tryptophan. For ruminants, barley is the third most readily degradable cereal behind oats and wheat. Due to its more rapid starch fermentation rate compared with corn, barley also provides a more synchronous release of energy and nitrogen, thereby improving microbial nutrient assimilation. As a result, feeding barley can reduce the need for feeding protected protein sources. However, this benefit is only realized if rumen acidity is maintained within an optimal range (e.g., > 5.8 to 6.0); below this range, microbial maintenance requirements and wastage increase. With a low pH, microbial endotoxines cause pro-inflammatory responses that can weaken immunity and shorten animal longevity. Thus, mismanagement in barley processing and feeding may make a tragedy from this treasure or pearl of cereal grains. Steam-rolling of barley may improve feed efficiency and post-rumen starch digestion. However, it is doubtful if such processing can improve milk production and feed intake. Due to the need to process barley less extensively than other cereals (as long as the pericarp is broken), consistent and global standards for feeding and processing barley could be feasibly established. In high-starch diets, barley feeding reduces the need for capacious small intestinal starch assimilation, subsequently reducing hindgut starch use and fecal nutrient loss. With its nutritional exclusivities underlined, barley use will be a factual art that can either matchlessly profit or harm rumen microbes, cattle production, farm economics and the environment.

## Introduction

Barley (*Hordeum Spp.*) is a cereal derived from the annual grass Hordeum Vulgare. This multipurpose grain deserves a top place in the farm for feeding livestock. It is irreplaceable by any other grain in beef and dairy diets for producing capacious rumen microbial yields [1]. This review delineates the nutritional and commercial status of barley and critically describes opportunities for its optimum use by rumen microbes, host ruminants, farmers and the environment.

### World production and distribution of barley

In ranking of cereal crops conducted by the Food and Agriculture Organization of the United Nations [2], barley was ranked fourth in the world both in terms of quantity produced (136 million tons) and in area of cultivation (566,000 km²). In 1994 to 1995, world production of barley was estimated at 166 million metric tons (MMT) or about 30 % of corn. In 2009 and 2010, world production of barley was 152 and 124 MMT, respectively (Table 1). The top barley producing countries are Germany, France, Ukraine and Russia [3].

**Table 1:** Top barley producers in the world (MMT)

**Country**	**2009**	**2010**
Germany	12.3	10.4
France	12.9	10.1
Ukraine	11.8	8.5
Russia	17.9	8.4
Spain	7.4	8.2
Canada	9.5	7.6
Australia	7.9	7.3
Turkey	7.3	7.2
United Kingdom	6.8	5.3
United States	5.0	3.9
World Total	151.8	123.7

Source: Food and Agriculture Organization (2010).

During 2004, approximately 2000 kt of barley and wheat were used by livestock in Australia representing 60 % of all cereals fed [2]. Oats, sorghum, and triticale contributed only 20 %, 10 % and 10 %. About 40 % of the barley was fed to feedlot cattle, 34 % to dairy cows, 20 % to pigs, 6 % to grazing ruminants, and < 1 % to poultry. In Canada, barley is primary used in beef and dairy cattle diets although some finds its way into swine diets [4,5]. Barley makes up 40 % of feed grain usage, equivalent to 7.3 MMT compared with 5.4 MMT for corn [1,3,5]. The U.S. (1.8 MMT), Japan (1.1 MMT) and Saudi Arabia (0.6 MMT) are major importers of Canadian barley [1-3].

### Nutritional value of barley

**Figure 1:**
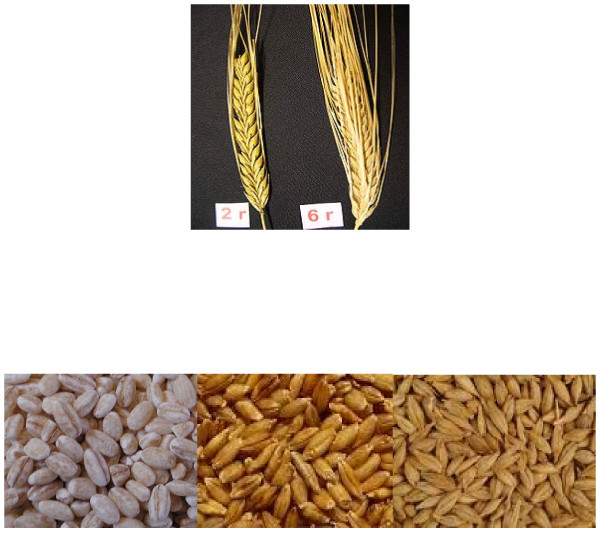
**Top: Varieties of two-rowed and six-rowed barley.** Bottom: Whole barley (right), naked or hull-less barley (middle) and pearled barley (left).

It is important to understand that barley is not just barley. Many types of barley exist and it is important to know the type of barley being fed and the consequences this might have in terms of nutrient content. There may be considerable dissimilarities, particularly in starch content and rumen fermentation patterns, between some barley cultivars [6]. Knowledge of such differences can help farmers select and feed the most appropriate varieties that optimize production without compromising rumen and host animal health. Examples of barley types are two-rowed vs. six rowed as well as whole, hulless and pearled barley (Figure 1).

The nutrient composition of barley compared with other cereal grains is shown in Table 2. In comparison with corn, barley has more protein, methionine and cysteine, lysine, and tryptophan. This information highlights the potential contribution of barley to meeting the protein requirements of high-producing ruminants [4,7]. In addition, in comparison with other cereal grains, barley contains the highest levels of neutral and acid detergent fiber and the lowest levels of starch and fat.

**Table 2:** Nutrient composition of barley compared with other cereals (g/kg)

**Nutrient (as fed)**	**Barley**	**Hull-less barley**	**Corn**	**Wheat**	**Sorghum**	**Rye**
Dry matter	880	880	880	880	880	880
Crude protein (CP)	115	132	88	135	110	121
Undegradable CP, g/kg CP	280	350	500	250	550	200
Neutral detergent fibre	181	120	108	118	161	180
Acid detergent fibre	60	20	30	40	90	100
Starch	570	650	720	770	720	620
Fat	19	20	38	22	29	15
Ash	23	19	14	17	18	19
Lysine	4.3	5.0	2.1	3.5	2.7	4.0
Methionine + Cysteine	4.2	5.6	3.0	5.1	3.0	3.6
Tryptophan	1.8	1.5	0.9	1.5	0.9	1.4
NE_L_, Mcal/kg	1.71	1.75	1.78	1.82	1.62	1.71

Data from Huntington (1997), NRC (2001). Net energy for lactation (NE_L_) of barley varies (e.g., 1.5-1.9 Mcal/kg) depending on dietary inclusion rate and processing method.

As shown in Table 3, barley is richest in potassium and vitamin-A among the common cereals. Barley grain contains five times more calcium than oats. With twice as much copper and molybdenum and > twice as much manganese, barley is superior to corn. However, barley is poorer in zinc compared with corn. The nutrients lacking in barley include vitamin C and vitamin B_12_. Noteworthy, few differences exist in nutrient composition between two-rowed and six-rowed barleys (Table 4).

**Table 3:** Mineral and vitamin content of the major cereal grains (g/kg of DM)

**Nutrient**	**Barley**	**Corn**	**Wheat**	**Oats**	**Sorghum**
Calcium	0.5	0.3	0.5	0.1	0.4
Phosphorous	3.5	3.2	4.4	4.1	3.4
Potassium	5.7	4.4	4.0	5.1	4.4
Magnesium	1.2	1.2	1.3	1.6	1.7
Sodium	0.1	0.1	0.1	0.2	0.1
Sulfur	1.5	1.1	1.4	2.1	1.4
Copper, ppm	5.3	2.5	6.5	8.6	4.7
Iron, ppm	59.5	54.5	45.1	94.1	80.8
Manganese, ppm	18.3	7.9	36.6	40.3	15.4
Selenium, ppm	-	0.14	0.05	0.24	0.46
Zinc, ppm	13.0	24.2	38.1	40.8	1.0
Cobalt, ppm	0.35	-	-	0.06	-
Molybdenum, ppm	1.16	0.60	0.12	1.70	-
Vit A, 1000 IU/kg	3.8	1.0	0.0	0.2	0.05
Vit E, 1000 IU/kg	26.2	25.0	14.4	15.0	12.0

NRC (1996, 2001).

**Table 4:** Average density and nutrient composition of North Dakota two-rowed and six-rowed barley varieties

**Nutrient**	**Two-row**	**Six-row**
Test weight, kg/bushel	48.4	46.2
Dry matter, g/kg	908	906
Neutral detergent fiber, g/kg	200	214
Acid detergent fiber, g/kg	62	66
Crude protein, g/kg	129	124
Calcium, g/kg	0.5	0.5
Phosphorous, g/kg	3.6	3.7
Magnesium, g/kg	1.4	1.4
Potassium, g/kg	5.4	5.4

Data from regional information during 1991–1997 [5,6].

**Figure 2:**
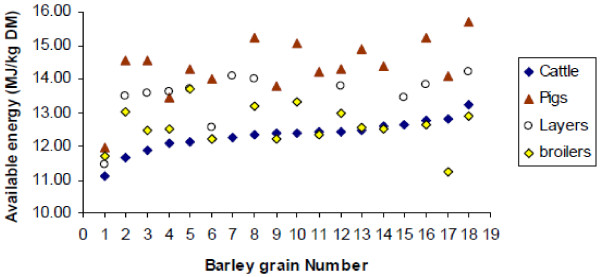
**Available energy for 18 samples of barley fed to livestock*ad libitum*(Adopted from**[10]**).**

Large differences exist among individual barley samples in terms of available energy and animal performance [8,9]. In an Australian assessment [10], pigs obtained greater energy from barley than other animals (Figure 2), whereas cattle utilized the energy in barley the least [10]. Correlations for the utilizable energy of barley between broilers and other animals were 0.77 for layers, 0.56 for pigs and 0.09 for cattle. The correlation between pigs and cattle was 0.71. These coefficients indicate significant differences among livestock in the digestive capacity of individual barleys. Some samples are more digestible by ruminants than pigs or poultry and indeed vice versa. Figure 2 shows that sample 1 was poorly digested by all animals. The useable energy of sample 4 was low for cattle and pigs, but medium for poultry. However, sample 5 provided low energy to cattle, high energy to poultry, and medium energy to pigs. The available energy of sample 17 was higher for cattle, lower for pigs, and much lower for poultry, whilst sample 18 generated more energy for cattle and pigs, low energy for broilers, and medium energy for layers.

Such versatilities in the energy value of barley originate from differential digestive systems and assimilative capacity between livestock species as well as disparities in chemical and physical properties of different barley samples [10]. Accordingly, assortment measures for breeding barley most suitable for different livestock can be developed. Barleys with low hull and fiber content, fragile cell walls, and thus low soluble arabinoxylans and ß-glucans and rapidly accessible starches are optimal for pigs. For poultry, samples with lower non-starch polysaccharides and thus lower viscosity, and low condensed tannins are greatly needed. On the other hand, for ruminants, cultivars with higher fiber and soluble arabinoxylans specifically with harder kernels to produce slower rumen starch degradation rates (i.e., low acidosis index) are preferred.

Near Infrared Reflectance Spectroscopy calibrations have been developed for premium grains in livestock programs to predict the available energy intakes for poultry, pigs, with other grain properties such as acidosis index. These calibrations help to monitor grains within barley breeding programs and to assign the most suitable grain samples to the appropriate livestock production system.

### Anti-nutritional factors in barley

Anti-nutritional factors occur in barley. A mycotoxin that grows on barley plants and barley is deoxynivalenol also known as vomitoxin. It is generated by a fusarium that grows on moist barley and wheat under humid conditions during the early heading stages. Nonetheless, evidence suggests no effects of vomitoxin on feed intake or milk production of cows.

### Feeding ruminants barley together with other grains and enzymes

**Figure 3:**
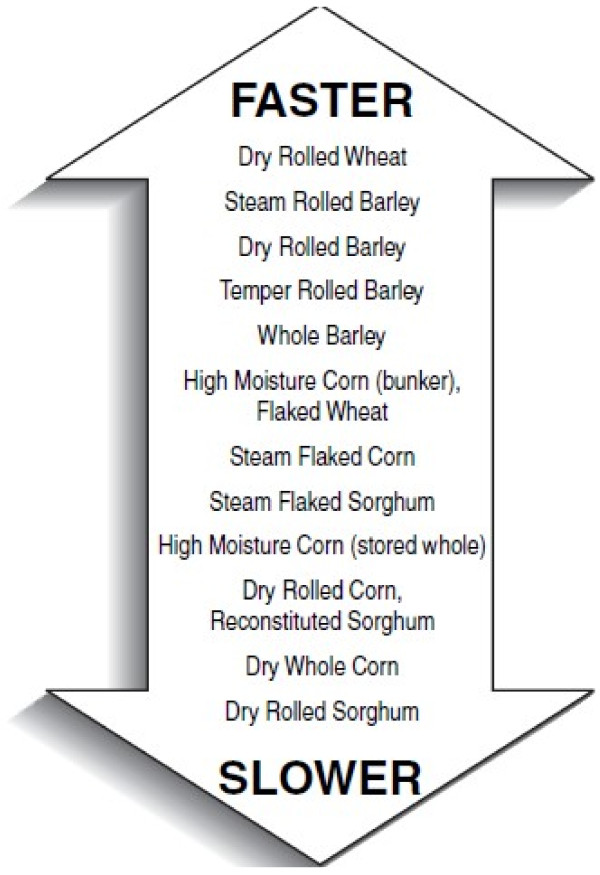
**Rumen dynamics of processed barley compared with other cereals.** Barley has one of the fastest degradation rates, preceded only by dry-rolled wheat.

Mixtures of grains offer advantages in beef and dairy cattle feeding [11]. This is due to their greater extent and rate of rumen starch fermentation [12,13]. Such blends can alleviate the rumen acidosis which usually occurs by feeding highly fermentable grains e.g., barley (Figure 3). Blending barley and corn, before processing/flaking, did not compromise feedlot cattle performance [14]. In grazing Jersey cows, replacing 50 % of corn with barley in concentrates increased milk production, suggesting positive associative effects of corn and barley [11]. More data on feeding combinations of different cereals are needed before clear-cut recommendations can be offered to the world ruminant industries. Adding xylanase-based fibrolytic enzymes to high concentrate (e.g., 950 g barley/kg of diet dry matter) diets improved feed efficiency without effects on daily gain and feed intake [15].

### Processing barley for beef and dairy cattle

Grain processing can affect the rate, extent and site of protein, fiber and starch digestion [16] (Figure 3, Table 5). Due to their inability to properly chew and break the husky kernels, whole barley cannot be fed to large ruminants [17]. As a result, barley is commonly rolled, tempered, steam-flaked, ground, roasted or pelleted [1]. While grinding is the most common and preferred technique to process barley for dairy cows in Iran [1,18], tempering, dry-rolling and steam-rolling are common in North America, Australia and Western Europe [19,20]. Tempering involves adding water for 24 hours prior to rolling to increase the moisture content of the barley up to 180 to 200 g/kg. Tempering results in fewer fine particles compared with dry rolling [21], which reduces the risk of rumen acidosis. Consequently, starch fermentation rate can decrease, thereby reducing the risks associated with a sharply-reduced rumen pH. As such, tempered barley, compared with dry rolled barley, improved milk yield by 5 %, feed efficiency by 10 %, and apparent digestibility of dry matter, neutral detergent fiber, acid detergent fiber, crude protein and starch by 6 %, 15 %, 12 %, 10 % and 4 %, respectively [22].

**Table 5:** ***In vivo*ruminal starch and protein degradation (% of intake) of differently proceed barley compared with wheat, corn and sorghum**^**1**^

**Processed grain**	**Rumen degradation, %**	**Post-rumen digestion, %**	**Total tract digestion, %**
Steam-rolled barley	84.6	13.6	98.2
Dry-rolled barley	80.7	13.7	94.3
Ground barley	88.0	10.5	98.5
Steam-rolled wheat	88.1	10.0	98.6
Dry-rolled wheat	88.3	9.9	98.2
Ground wheat	90.0	8.9	99.9
Steam-flaked corn	84.8	14.1	98.9
Dry-rolled corn	76.2 (35)^2^	16.2	92.4
Ground corn	49.5	44.0	93.5
Steam-flaked sorghum	78.4	19.6	98.0
Dry-rolled sorghum	59.8	26.1	87.2
Ground sorghum	70	15.4	91.0

^1^Data obtained from Nikkhah et al. (2004), Nikkhah and Ghorbani (2003), Huntington (1997), Herrera-Saldana et al. (1990) (51–55).

^2^The value within parenthesis shows dramatic variation.

Aggressive and high-pressure exposure to heat may reduce the degradation rate of barley [23]. This reduction is important *in vivo*, especially directly after feeding when rumen fermentation peaks. Such moderated barley degradation rates can improve feed efficiency likely via increased rumen pH and attenuated rumen acidosis during fermentation peaks as well as increased small intestinal escape or partially-digested starch assimilation [24]. Likewise, flame roasting of barley reduced dry matter and crude protein rumen degradation despite no effects on total tract digestibilities [25]. Feeding roasted barley instead of rolled barley twice a day improved milk yield by 3 kg [25]. Nonetheless, *in vivo* actual data (versus *in vitro* and *in situ* estimates) on post-rumen and especially small intestinal digestion of protein and starch from differently processed barley in high-producing ruminants are greatly limited.

Feeding yearling steers steam-rolled barley instead of high moisture corn in diets with 650 g grain, 160 g forage, 50 g supplement and 140 g potato residues per kg of diet did not affect weight gain, but decreased dry matter intake cubically with increased levels of barley [26]. In finishing diets with 840 g grain, 120 g alfalfa haylage and 40 g supplement per kg of diet, dry-rolled barley and corn affected cattle performance, carcass properties, and the incidence of digestive disorders similarly [27]. Replacing dry-rolled corn with tempered barley in 60 g/kg forage finishing diets resulted in no differences in intake and weight gain in response to different ratios of the two grains [28]. However, steers fed the blend of grains had greater carcass weights, yield grades, and 12th rib fat than did steers fed single grains. These data suggest more efficient uses of barley when fed in combination with corn rather than when fed alone.

Steam-rolled barley was similar to steam-rolled corn in affecting milk yield of lactating cows [29]. This was also the case in complete mixed cubed diets [30], with dry-rolled barley versus ground corn [30,31], or with both grains in the ground form [32,33]. Dry-rolled barley successfully replaced the high-energy dry-rolled grain sorghum with respect to milk yield, and tended to improve feed efficiency [34]. Dry rolled barley and ground corn with and without bovine somatotropin (bST) similarly affected bST response, milk production, somatic cell count, and cow weight [35]. However, slight declines in milk production and feed intake were reported for barley versus corn fed cattle [36]. This could be due to overly reduced rumen pH and depressed fiber digestion and the supply of milk precursors under suboptimal circumstances. With prudent and more moderate uses in dairy diets, ground barley has proved superior to ground broomcorn and as competent as steam-flaked broomcorn in maintaining feed intake and milk production [37] (Table 6). These findings emphasize the science-based experience that dietary inclusion rate of barley requires more deserving thoughts for optimal rumen function and ruminant production and welfare [1,18,38].

**Table 6:** Production, digestion, and metabolism of mid-lactation Holstein cows fed ground versus steam-rolled barley-based total mixed rations containing corn silage and alfalfa hay

**Level of barley**	**26 % barley**		**32.5 % barley**	
**Processing method**	**Ground**	**Rolled**	**Ground**	**Rolled**
Dietary starch, %	18.0	18.0	26.8	26.8
Dietary neutral detergent fiber, %	38.0	38.0	34.3	34.3
Dietary acid detergent fiber, %	24.1	24.1	19.0	19.0
Dietary crude protein, %	16.1	16.1	18.9	18.9
Dry matter intake, kg/d	23.5	23.9	24.8	24.4
Fat corrected milk, kg/d	27.8	28.6	36.1	37.7
Milk fat yield, kg/d	1.07	1.07	1.29	1.37
Milk protein yield, kg/d	0.83	0.85	1.07	1.10
Rumen pH	6.7	6.6	5.7	5.7
Chewing time, min/d	-	-	820	803
Total tract dry matter digestibility %	69.0	69.0	-	-

Nikkhah (2011); Sadri et al. (2007); Soltani et al. (2009) (18,37,39).

Based on NRC recommendations [7], dairy diets should contain 25 % to 28 % neutral detergent fiber, 75 % of which must be supplied by forages. This is needed for adequate chewing and healthy rumen function, and to prevent milk fat depression and laminitis [24]. Barley-based diets usually supply greater amounts of neutral detergent fiber than corn-based diets. However, due to the inadequate effectiveness of the neutral detergent fiber of barley in stimulating chewing and ensalivation as well as the greater degradation rate of barley than corn, barley-fed cows require greater effective forage fiber than corn fed cows [29]. Normally, rumen cellulolytic bacteria numbers are sufficiently maintained under pH > 6.0. Thus, so long as barley feeding does not lower rumen pH below 5.8 to 6.0, it can replace the more expensive corn in dairy diets.

Recent findings compellingly suggest that finely ground barley is not inferior to the more expensive steam-rolled barley if dietary barley inclusion rate is kept sensibly moderate at ≤ 300 g/kg of diet dry matter [39,40] (Table 6). Even at 350 g/kg barley, except for a modest improvement in feed efficiency, milk production and dry matter intake were similar between ground and steam-rolled barley fed cows [18].

Overfeeding barley is an easy shortcut to rumen acidosis and triggered pro-inflammatory responses of depressed immune function [41,42]. Feeding > 35 % barley/kg of dietary dry matter is under no circumstances recommended. Thus, whilst barley is a matchless source of rapidly released energy for efficient rumen microbial mass and volatile fatty acid yields, its dietary use must be an art to allow such benefits to become a reality in optimizing production and health concomitantly [1]. As much as being the pearl of cereals, indispensable for persistent peaks in beef and dairy production, improper feeding of no other grain can be as much economically and environmentally devastating as barley [43,44].

### Rumen physiology and health aspects of barley feeding

Cows fed overly high amounts of rapidly fermentable starches such as barley are very likely to experience periods of subacute rumen acidosis which can increase the incidence of laminitis [45,46]. High levels of ground cereals are also thought to predispose cattle to lameness, resulting from acidosis. These challenges occur mostly because barley, regardless of processing technique, has a much greater extent of rumen fermentation and higher fermentation rate than other processed grains, preceded only by dry-rolled wheat grain (Table 5, Figure 3). Recent evidence suggests that with optimal barley inclusion rate in dairy rations, ground barley can be as palatable and effectively utilized as steam-processed barley [1,18] (Table 6). Thus, pragmatically, it is not grinding that is problematic, but it is rather the very high dietary levels of barley that introduces serious challenges to the rumen and cow metabolism and immunity [1,41].

**Figure 4:**
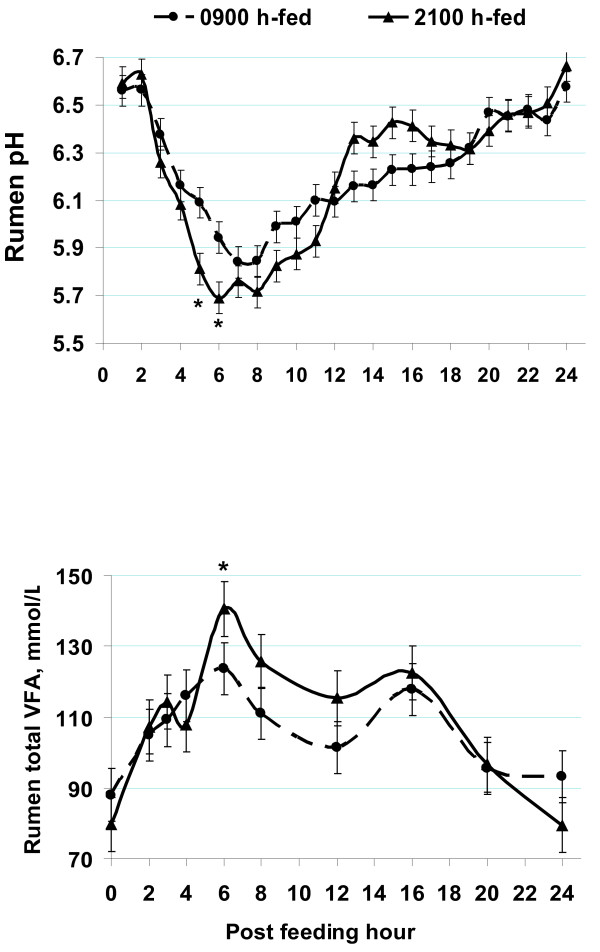
**Circadian and post-feeding rumen pH and total volatile fatty acid patterns in 8 cows fed a barley-grain based high-concentrate mixed ration once daily at either 0900 h or 2100 h**[38]**,**[48]**.**

**Figure 5:**
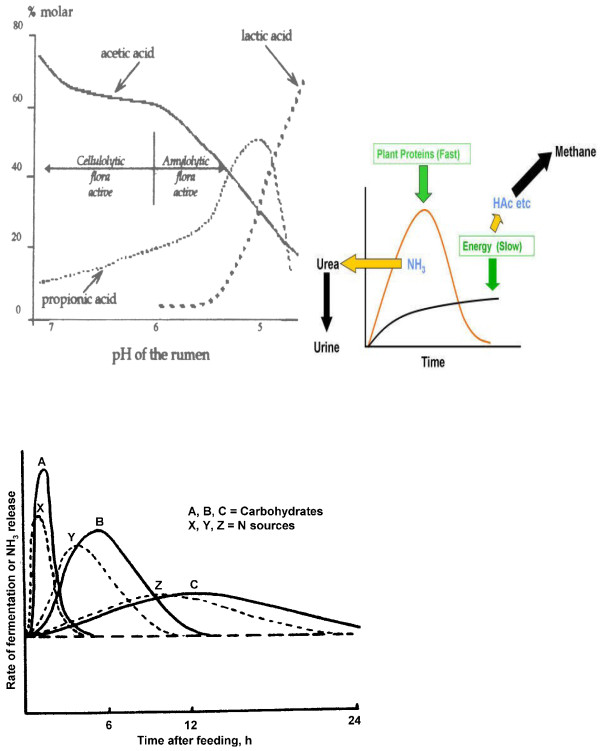
**Top right: The slower rate of dietary energy vs. protein fermentation.** Top left: Relationships among rumen pH, differential volatile fatty acids and lactate concentrations and prevalence of cellulolyric versus amylolytic bacteria. Bottom: Rumen release of rapidly (A, X), moderately (B, Y) and slowly (C, Z) degradable carbohydrates and nitrogen fractions over time for microbial mass yield. The AX and BY curves would represent post-feeding fermentation patterns of barley and corn respectively ([1,5,12,54,55]). Increased asynchrony of carbohydrate and protein release and prolonged rumen acidosis can make a tragedy from the treasure barley.

As illustrated in Figures 4 and 5, rumen fermentation possesses circadian patterns in pH and volatile fatty acid concentrations that depend on feed delivery and feeding behavior [47,48]. As such, most dramatic fluctuations occur around feeding and shortly after when the rumen receives a considerable amount of substrate.

A common challenge in optimizing rumen fermentation is the asynchrony in fermentation rate and patterns of protein and energy [1,49-55] (Figure 5). Proteins and carbohydrates have rapidly, moderately, and slowly degradable fractions and each of these nourish specific microbial populations. In addition, proteins are usually degraded more rapidly than carbohydrates upon feeding (Figure 5). This means that the maximum rumen energetic potential is reached when proteins have already gone through their maximum degradation. Thus, loss of nitrogen and energy as ammonia, methane and carbon dioxide would result.

**Figure 6:**
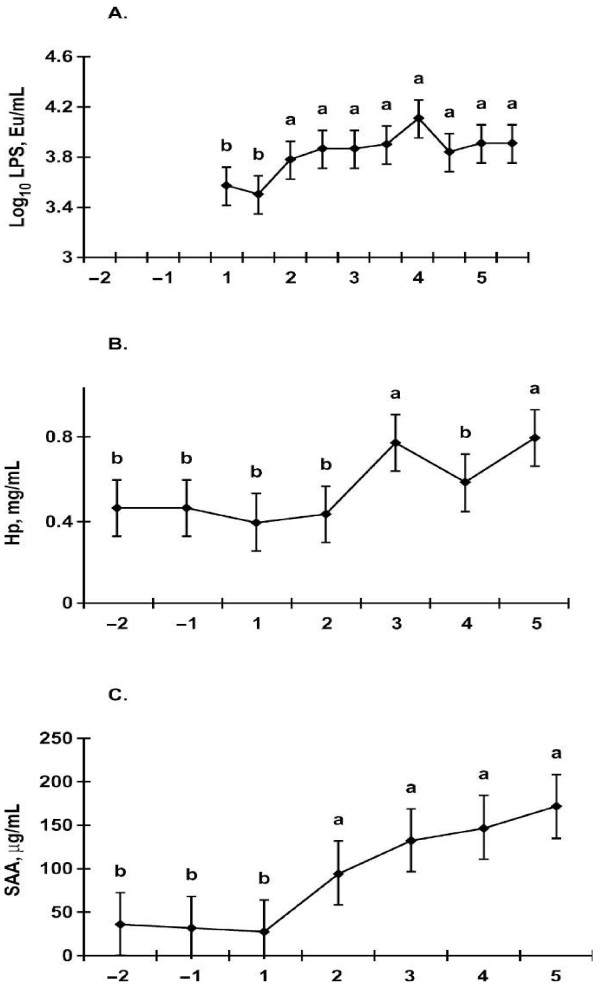
**Ruminal lipopolysaccharides (LPS, log10 endotoxin units/mL; Figure A), haptoglobin (Hp; Figure B), and serum amyloid-A (SAA; Figure C) in steers fed chopped alfalfa hay only (days −2 and −1) and barley-wheat based pelleted concentrate (days 1 to 5).** Diet 1 = 4 kg of barley-wheat pellets and 6 kg of chopped alfalfa hay offered daily; Diet 2 = 5 kg of barley-wheat pellets and 5 kg of chopped alfalfa hay offered daily; Diet 3 = 6 kg of barley-wheat pellets and 4 kg of chopped alfalfa hay offered daily. Different letters declare statistical significance. Daily duration of time at which pH was below 5.6 (as an indicator of subacute rumen acidosis) was 42, 117, and 134 min/d for Diets 1, 2, and 3, respectively [42].

Feeding barley-based diets is expected to alter fermentation patterns (Figure 5), such that an earlier energy fermentation peak would occur to reduce the asynchrony and improve substrate incorporation into the microbial mass. Such shifted fermentation patterns can optimize energy efficiency and milk biosynthesis, and reduce methane, ammonia and urinary nitrogen outputs [18]. However, due to its highly degradable nature in the rumen, regardless of processing method, barley must not be overfed (e.g., < 35 % of diet dry matter) (Tables 5,6) [1,18,39,40]. Under rapid fermentation of the overfed barley starch, rumen pH will fall and persist below 5.8 where rumen acidosis will govern. The incidence of rumen acidosis in large herds can have detrimental consequences on feed efficiency and economical sustainability [50]. Dramatic and persistent acidic environments will coexist with, and further result in, increased lactic acid production. Lactic acid has a lower pKa than the volatile fatty acids (3.8 vs. 4.8). At lower pH, greater proportions of lactic acid will occur in undissociated forms [43,50], the accumulation of which plus that of volatile fatty acids will progressively interfere with efficient acid absorption, thus prolonging rumen acidosis and exacerbating the problem. Under such acidotic conditions, microbial mass yield will drop noticeably and bacteria will lyse, which will cause endotoxin release and trigger systemic pro-inflammatory responses [51]. This is evident in elevated circulating levels of haptoglobin and serum amyloid-A indicative of rumen acidosis in barley fed cattle [42] (Figure 6). Therefore, rumen acidosis can weaken cattle immunity and depress productivity and thereby threaten farm economics and sustainability.

## Conclusions and implications

Barley grain is known for its thick fibrous coat, high content of ß-glucans and less complicated starch granules. With about 150 MMT of annual yield, world production of barley is about 30 % of corn. Universally, barley is typically cheaper and less demanded by non-ruminants and humans than corn and wheat. Besides greater protein, barley is richer in methionine, lysine, cysteine, and tryptophan than corn. Barley is considered highly degradable in the rumen. Owing to its more rapid and extensive rumen starch and nitrogen fermentation compared with ground corn, barley may provide more synchronous energy and nitrogen release, which can improve microbial and host nutrient assimilation. Proper barley feeding management may reduce expensive undegradable protein requirements. Conversely, with improper dietary inclusion rate and processing, no other grain can as easily be a shortcut to prolonged rumen acidosis, microbial endotoxin release, pro-inflammatory responses, and suppressed immune function as barley. Due to the need to process barley less extensively than corn, sorghum or wheat (as long as the pericarp is broken), establishing consistent and global standards for feeding and processing could be more feasible for barley than other grains. Feeding barley to modern ruminants must be a factual art that will matchlessly profit or otherwise dramatically impair rumen microbes, host health and production, farm economics, and the environment. Optimal dietary inclusion rates of barley are where global tragedies could be well avoided by a treasure.

## Abbreviations

ADF, Acid Detergent Fiber; BG, Barley Grain; CG, Corn grain; CP, Crude Protein; DM, Dry Matter; NDF, Neutral Detergent Fiber; SARA, Subacute Rumen Acidosis; TMR, Total Mixed Ration; VFA, Volatile Fatty Acids.

## Competing interests

The author declares no competing interests.

## Author details

Doctor Akbar Nikkhah, PH.D., is Highly Distinguished Professor of Science and Ruminant Nutrition, Highly Distinguished Mentor of Science Education and Dissemination, and Highly Distinguished Science Composer/Speaker currently in the Department of Animal Sciences, University of Zanjan in Iran; and Highly Distinguished Elite-Generating Scientist of the National Elite Foundation in Iran. He is also Highly Distinguished Global Peace Leader.
